# Structure-Activity Relationships of Insect Defensins

**DOI:** 10.3389/fchem.2017.00045

**Published:** 2017-07-12

**Authors:** Johannes Koehbach

**Affiliations:** School of Biomedical Sciences, University of Queensland St. Lucia, QLD, Australia

**Keywords:** insect peptides, cysteine-stabilized, antimicrobial activity, drug design, antibiotic drug discovery

## Abstract

Insects make up the largest and most diverse group of organisms on earth with several million species to exist in total. Considering the sheer number of insect species and the vast variety of ways they interact with their environment through chemistry, it is clear that they have significant potential as a source of new lead molecules. They have adapted to a range of ecological habitats and exhibit a symbiotic lifestyle with various microbes such as bacteria and fungi. Accordingly, numerous antimicrobial compounds have been identified including for example defensin peptides. Insect defensins were found to have broad-spectrum activity against various gram-positive/negative bacteria as well as fungi. They exhibit a unique structural topology involving the complex arrangement of three disulfide bonds as well as an alpha helix and beta sheets, which is known as cysteine-stabilized αβ motif. Their stability and amenability to peptide engineering make them promising candidates for the development of novel antibiotics lead molecules. This review highlights the current knowledge regarding the structure-activity relationships of insect defensin peptides and provides basis for future studies focusing on the rational design of novel cysteine-rich antimicrobial peptides.

## Introduction

Peptides are known to play pivotal roles in many physiological functions and besides their action as signaling molecules they are crucial for the interaction with other organisms. This includes for example antimicrobial peptides (AMP) that represent an important part of the organism's defense machinery or peptide toxins as part of venom cocktails (Brogden et al., 2003; Favreau et al., 2006; Aili et al., 2014). AMPs are a diverse class of naturally occurring compounds that have been identified in a variety of organisms, from invertebrates to vertebrates including humans (Shafee et al., 2017). In particular insects are known for their immune system that has evolved a complex arrangement of constitutive and inducible AMPs that are used to defend against invading microorganisms (Kingsolver et al., 2013) and allow a symbiotic lifestyle with various microbes (Douglas, 2015). With regard to the quest for novel antimicrobial agents to target multidrug resistant pathogens such insect AMPs are promising starting points for antibiotic drug development approaches (Ageitos et al., 2016; Mahlapuu et al., 2016). The variety of these peptides, both in terms of structure as well as activity reflects the unique diversity of insect species. Insects are the largest and most diverse group of living organisms on earth (Hellmann and Sanders, 2007). Approximately, 950,000 species are described to date (Berenbaum and Eisner, 2008) and around 4,000,000 insect species are estimated to exist in total (May, 2000). Considering the sheer number of insect species and the vast variety of ways they interact with their environment through chemistry, it is clear that they have significant potential as a source of new lead molecules (Dossey, 2010). In particular insect AMPs have recently attracted increased attention with regard to their possible medical as well as agrochemical applications (Yi et al., 2014; Tonk and Vilcinskas, 2017).

Bioactive peptides are promising novel drug leads that may fill the gap between small molecules and larger biologicals. This is reflected by a multitude of recent peptide discovery and development approaches (Craik et al., 2013). However, their use as therapeutic lead molecules is challenged by their typically poor stability and lack of oral bioavailability (Adessi and Soto, 2002; Otvos and Wade, 2014). This is often due to the linear nature of peptides that not only exhibit free ends but multiple cleavage sites that are readily recognized by enzymes that degrade peptide chains into inactive fragments or single amino acids. The presence of multiple disulfide bonds resulting in more compact structures typically increases stability and bioavailability. Several naturally-occurring disulfide-rich peptide families have been described such as plant cyclotides, knottins, conotoxins, or relaxin family peptide hormones. These cysteine-stabilized peptides are exhibiting a well-defined three-dimensional structure making them of particular interest for structure-activity relationship studies (Huang et al., 2010; Clark et al., 2011; Akondi et al., 2014; Patil et al., 2016). Due to their unique three-dimensional topologies, a wide range of activities and amenability to chemical synthesis they have been shown to represent valuable structural templates for peptide engineering (Carstens et al., 2011; Poth et al., 2013; Kintzing and Cochran, 2016). Within insects such a class of disulfide-rich peptides is known as “insect defensins” (Lambert et al., 1989). They exhibit a complex three-dimensional pattern, which is referred to as cysteine stabilized αβ motif (CSαβ). Importantly, as part of the immune system these peptides were found to have a broad spectrum of antimicrobial activities against bacteria, fungi and other parasites. This review will (i) highlight the current knowledge regarding structure-activity relationships of insect defensins and aims to (ii) provide a basis for future rational design of novel cysteine-rich AMPs.

## Exploring the diversity of cysteine-rich insect antimicrobial peptides

**Figure 1:**
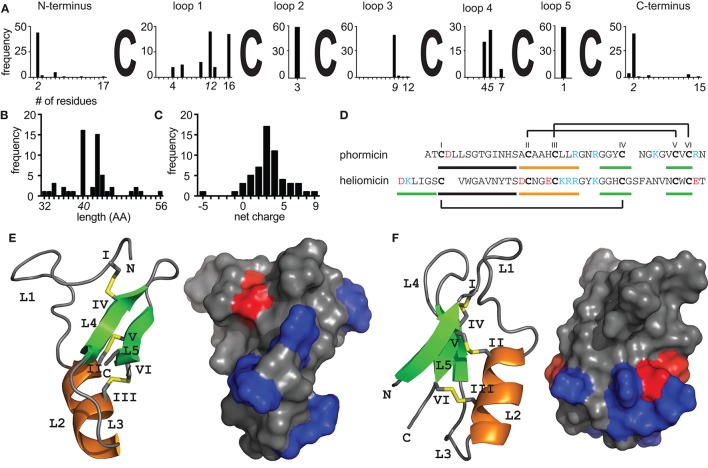
Structural diversity of insect defensins. Sequence analysis of 57 peptides (Table 1) illustrating the diversity of insect defensins. **(A)** All sequences share a conserved pattern of six cysteine residues. Intercysteine loop 2 (*n* = 3) and loop 5 (*n* = 1) are fully conserved across all peptides and loop 1 shows the overall highest sequence variability (*n* = 4–16). The minimal, maximal, and most abundant (italic font) length of individual sequence stretches are indicated. **(B)** The length distribution shows that insect defensins are between 32 and 52 residues long, with the majority (54%) being 40 or 43 residues. **(C)** Insect defensins are typically cationic with reported charges varying between −5 and +8 and the majority (49%) of peptides holding a +3 or +4 net charge. **(D)** Sequences of the antibacterial phormicin and the antifungal heliomicin showing the disulfide connectivity of insect defensins. Stretches that form part of the α-helix (orange) and β-sheets (green) are indicated. Solution NMR structures of **(E)** phormicin (PDB: 1ICA) and **(F)** heliomicin (PDB: 1I2U) showing the cysteine-stabilized αβ motif. Secondary structural elements, i.e., α-helix (orange), β-sheets (green) and disulfide bonds (yellow) are highlighted, cysteines (roman numerals) and loops (L) are indicated. Surface representations show negatively (red) and positively (blue) charged residues, demonstrating the overall cationic character of the surface of the peptides.

Given the sheer number of different insect species, it is not surprising that a variety of different AMPs can be found within these organisms. The ability of insects to adapt to almost every terrestrial ecosystem and their exposure to a variety of pathogens is reflected in the expression of different types and numbers of AMPs in individual species (Vilcinskas, 2013). However, the discovery of bioactive peptides from such small organisms by classical mass spectrometry based peptidomics approaches is facing serious challenges. This is due to highly complex samples that are limited in quantity and bioactive compounds present in trace amounts only that can easily be overlooked or only partially be identified (Wiese et al., 2006; Ueberheide et al., 2009). To fully unveil the diversity of peptides from such biological samples new advances in analytical chemistry, nucleotide sequencing and high-throughput drug screening are essential to aid in the discovery of novel AMPs. Indeed, refined methodologies that combine classical chemical analysis with bioinformatics workflows have proven useful to harness the variety of peptides and expand the knowledge of natural product peptidomes (Koehbach and Jackson, 2015). Though, to date the majority of identified defensin sequences was retrieved using either mass spectrometry based characterization of insect hemolymphs or cDNA cloning (see references in Table 1). Recent studies that describe the use of transcriptomes and genomes as valuable source of novel defensin sequences are significantly expanding the number of identified peptides per single study (Gruber and Muttenthaler, 2012; Poppel et al., 2015) and provide new insights into defensin diversity. Although nucleotide based peptide discovery provides additional information about the biosynthetic origin of peptides it lacks information regarding potential post-translational modification such as C-terminal amidation. Defensins are embedded in larger precursor molecules that consist of an ER signal peptide, a propeptide domain that precedes the mature peptide domain and ends with a conserved dibasic cleavage motif (Gruber and Muttenthaler, 2012). Mature peptides are typically around 40 residues long and carry an overall positive net charge with infrequent reports of anionic examples (Figures 1B,C; Wen et al., 2009; Xu et al., 2016).

**Table 1:** Naturally occurring insect defensin peptides with reported antimicrobial activity.

**Peptide^a^ (Organism)**	**Length (AA)**	**Net charge**	**Activity^b^**			**References**
			**G+**	**G−**	**F**	
Heliomicin^c^ (*Heliothis virescens*)						Lamberty et al., 1999
DKLIGS$\underset{\_}{\text{C}}$VWGAVNYTSD$\underset{\_}{\text{C}}$NGE$\underset{\_}{\text{C}}$KRRGYKGGH$\underset{\_}{\text{C}}$GSFANVN$\underset{\_}{\text{C}}$W$\underset{\_}{\text{C}}$ET	44	+1	–	–	X	
ARD1^c^ (*Archaeoprepona demophon*)						Landon et al., 2004
DKLIGS$\underset{\_}{\text{C}}$VWGAVNYTSN$\underset{\_}{\text{C}}$NAE$\underset{\_}{\text{C}}$KRRGYKGGH$\underset{\_}{\text{C}}$GSFANVN$\underset{\_}{\text{C}}$W$\underset{\_}{\text{C}}$ET	44	+2	n.d.	n.d.	X	
GmDefensin, GmDef-like(*Galleria mellonella*)						Lee et al., 2004; Cytrynska et al., 2007
DTLIGS$\underset{\_}{\text{C}}$VWGATNYTSD$\underset{\_}{\text{C}}$NAE$\underset{\_}{\text{C}}$KRRGYKGGH$\underset{\_}{\text{C}}$GSFLNVN$\underset{\_}{\text{C}}$W$\underset{\_}{\text{C}}$E	43	0	–	–	X	
DKLIGS$\underset{\_}{\text{C}}$VWGATNYTSD$\underset{\_}{\text{C}}$NAE$\underset{\_}{\text{C}}$KRRGYKGGH$\underset{\_}{\text{C}}$GSFWNVN$\underset{\_}{\text{C}}$W$\underset{\_}{\text{C}}$EE	44	0	X	–	X	
Gallerimycin(*Galleria mellonella*)						Schuhmann et al., 2003
GVTITVKPPFPG$\underset{\_}{\text{C}}$VFYE$\underset{\_}{\text{C}}$IAN$\underset{\_}{\text{C}}$RSRGYKNGGY$\underset{\_}{\text{C}}$TING$\underset{\_}{\text{C}}$Q$\underset{\_}{\text{C}}$LR	42	+4	–	–	X	
Drosomycin^c^ (*Drosophila melanogaster*)						Fehlbaum et al., 1994
D$\underset{\_}{\text{C}}$LSGRYKGP$\underset{\_}{\text{C}}$AVWDNET$\underset{\_}{\text{C}}$RRV$\underset{\_}{\text{C}}$KEEGRSSGH$\underset{\_}{\text{C}}$SPSLK$\underset{\_}{\text{C}}$W$\underset{\_}{\text{C}}$EG$\underset{\_}{\text{C}}$	44	+1	–	–	X	
Termicin^c^ (*Pseudocanthotermes spiniger*)						Lamberty et al., 2001b
A$\underset{\_}{\text{C}}$NFQS$\underset{\_}{\text{C}}$WAT$\underset{\_}{\text{C}}$QAQHSIYFRRAF$\underset{\_}{\text{C}}$DRSQ$\underset{\_}{\text{C}}$K$\underset{\_}{\text{C}}$VFVRG^*^^d^	36	+4	–	–	X	
Defensin(*Aeshna cyanea*)						Bulet et al., 1992
GFG$\underset{\_}{\text{C}}$PLDQMQ$\underset{\_}{\text{C}}$HRH$\underset{\_}{\text{C}}$QTITGRSGGY$\underset{\_}{\text{C}}$SGPLKLT$\underset{\_}{\text{C}}$T$\underset{\_}{\text{C}}$YR	38	+3	X	–	n.d.	
Insect defensin A (phormicin)^c^, B(*Phormia terranovae*)						Lambert et al., 1989
AT$\underset{\_}{\text{C}}$DLLSGTGINHSA$\underset{\_}{\text{C}}$AAH$\underset{\_}{\text{C}}$LLRGNRGGY$\underset{\_}{\text{C}}$NGKGV$\underset{\_}{\text{C}}$V$\underset{\_}{\text{C}}$RN	40	+3	X	X	X	
Sapecin A^c^, B, C(*Sarcophaga peregrina*)						Matsuyama and Natori, 1988; Yamada and Natori, 1993
AT$\underset{\_}{\text{C}}$DLLSGTGINHSA$\underset{\_}{\text{C}}$AAH$\underset{\_}{\text{C}}$LLRGNRGGY$\underset{\_}{\text{C}}$NGKAV$\underset{\_}{\text{C}}$V$\underset{\_}{\text{C}}$RN	40	+3	X	–	–	
LT$\underset{\_}{\text{C}}$EIDRSL$\underset{\_}{\text{C}}$LLH$\underset{\_}{\text{C}}$RLKGYLRAY$\underset{\_}{\text{C}}$SQQKV$\underset{\_}{\text{C}}$R$\underset{\_}{\text{C}}$VQ	34	+4				
AT$\underset{\_}{\text{C}}$DLLSGIGVQHSA$\underset{\_}{\text{C}}$ALH$\underset{\_}{\text{C}}$VFRGNRGGY$\underset{\_}{\text{C}}$TGKGI$\underset{\_}{\text{C}}$V$\underset{\_}{\text{C}}$RN	40	+3				
Tenecin 1(*Tenebrio molitor*)						Moon et al., 1994
VT$\underset{\_}{\text{C}}$DILSVEAKGVKLNDAA$\underset{\_}{\text{C}}$AAH$\underset{\_}{\text{C}}$LFRGRSGGY$\underset{\_}{\text{C}}$NGKRV$\underset{\_}{\text{C}}$V$\underset{\_}{\text{C}}$R	43	+4	X	–	n.d.	
Defensin(*Allomyrina dichotoma*)						Miyanoshita et al., 1996
VT$\underset{\_}{\text{C}}$DLLSFEAKGFAANHSL$\underset{\_}{\text{C}}$AAH$\underset{\_}{\text{C}}$LAIGRRGGS$\underset{\_}{\text{C}}$ERGV$\underset{\_}{\text{C}}$I$\underset{\_}{\text{C}}$RR	43	+3	X	–	n.d.	
AaeDef A(*Aedes aegypti*)						Lowenberger et al., 1995
AT$\underset{\_}{\text{C}}$DLLSGFGVGDSA$\underset{\_}{\text{C}}$AAH$\underset{\_}{\text{C}}$IARGNRGGY$\underset{\_}{\text{C}}$NSKKV$\underset{\_}{\text{C}}$V$\underset{\_}{\text{C}}$RN	40	+3	X	X	n.d.	
AalDefD(*Aedes albopictus*)						Gao et al., 1999
AT$\underset{\_}{\text{C}}$DLLSGFGVGDSA$\underset{\_}{\text{C}}$AAH$\underset{\_}{\text{C}}$IARGNRGGY$\underset{\_}{\text{C}}$NSKKV$\underset{\_}{\text{C}}$V$\underset{\_}{\text{C}}$PI	40	+2	–	X	n.d.	
AgaDef 1 (Def-AAA)^c^, 2(*Anopheles gambiae*)						Richman et al., 1996
AT$\underset{\_}{\text{C}}$DLASGFGVGSSL$\underset{\_}{\text{C}}$AAH$\underset{\_}{\text{C}}$IARRYRGGY$\underset{\_}{\text{C}}$NSKAV$\underset{\_}{\text{C}}$V$\underset{\_}{\text{C}}$RN	40	+4	X	–	–	
QLKNLA$\underset{\_}{\text{C}}$VTNEGPKWANTY$\underset{\_}{\text{C}}$AAV$\underset{\_}{\text{C}}$HMSGRGAGS$\underset{\_}{\text{C}}$NAKDE$\underset{\_}{\text{C}}$V$\underset{\_}{\text{C}}$SMT	45	+1	X	–	–	
Smd 1,2(*Stomoxys calcitrans*)						Lehane et al., 1997
AAKPMGIT$\underset{\_}{\text{C}}$DLLSLWKVGHAA$\underset{\_}{\text{C}}$AAH$\underset{\_}{\text{C}}$LVLGDVGGY$\underset{\_}{\text{C}}$TKEGL$\underset{\_}{\text{C}}$V$\underset{\_}{\text{C}}$KE	46	0	n.d.	–	n.d.	
AT$\underset{\_}{\text{C}}$DLLSMWNVNHSA$\underset{\_}{\text{C}}$AAH$\underset{\_}{\text{C}}$LLLGKSGGR$\underset{\_}{\text{C}}$NDDAV$\underset{\_}{\text{C}}$V$\underset{\_}{\text{C}}$RK	40	+1	n.d.	X	n.d.	
GmDef A^d^ (*Glossina morsitans*)						Boulanger et al., 2002
VT$\underset{\_}{\text{C}}$NIGEWV$\underset{\_}{\text{C}}$VAH$\underset{\_}{\text{C}}$NSKSKKSGY$\underset{\_}{\text{C}}$SRGV$\underset{\_}{\text{C}}$Y$\underset{\_}{\text{C}}$TN	33	+3	X	–	n.d.	
RprDef A, B, C(*Rhodnius prolixus*)						Lopez et al., 2003
AT$\underset{\_}{\text{C}}$DLFSFRSKWVTPNHAA$\underset{\_}{\text{C}}$AAH$\underset{\_}{\text{C}}$LLRGNRGGR$\underset{\_}{\text{C}}$KGTI$\underset{\_}{\text{C}}$H$\underset{\_}{\text{C}}$RK	43	+7				
AT$\underset{\_}{\text{C}}$DLLSFRSKWVTPNHAG$\underset{\_}{\text{C}}$AAH$\underset{\_}{\text{C}}$LLRGNRGGH$\underset{\_}{\text{C}}$KGTI$\underset{\_}{\text{C}}$H$\underset{\_}{\text{C}}$RK	43	+6	X	–	–	
AT$\underset{\_}{\text{C}}$DLFSFRSKWVTPNHAG$\underset{\_}{\text{C}}$AAH$\underset{\_}{\text{C}}$IFLGNRGGR$\underset{\_}{\text{C}}$VGTV$\underset{\_}{\text{C}}$H$\underset{\_}{\text{C}}$RK	43	+5				
Lucifensin^c^ [*Lucilia sericata, L. cuprina* (II)]						Cerovsky et al., 2010; El Shazely et al., 2013
AT$\underset{\_}{\text{C}}$DLLSGTGVKHSA$\underset{\_}{\text{C}}$AAH$\underset{\_}{\text{C}}$LLRGNRGGY$\underset{\_}{\text{C}}$NGRAI$\underset{\_}{\text{C}}$V$\underset{\_}{\text{C}}$RN	40	+4	X	n.d.	n.d.	
AT$\underset{\_}{\text{C}}$DLLSGTGIKHSA$\underset{\_}{\text{C}}$AAH$\underset{\_}{\text{C}}$LLRGNRGGY$\underset{\_}{\text{C}}$NGRAI$\underset{\_}{\text{C}}$V$\underset{\_}{\text{C}}$RN	40	+4	X	n.d.	n.d.	
LSerDef 3, 4, 6, 7(*Lucilia sericata*)						Poppel et al., 2015
AT$\underset{\_}{\text{C}}$DLLSGTGANHSA$\underset{\_}{\text{C}}$AAH$\underset{\_}{\text{C}}$LLRGNRGGY$\underset{\_}{\text{C}}$NSKAV$\underset{\_}{\text{C}}$V$\underset{\_}{\text{C}}$RN	40	+3	X	–	n.d.	
LT$\underset{\_}{\text{C}}$NIDRSF$\underset{\_}{\text{C}}$LAH$\underset{\_}{\text{C}}$LLRGYKRGF$\underset{\_}{\text{C}}$TVKKI$\underset{\_}{\text{C}}$V$\underset{\_}{\text{C}}$RH	34	+6				
GT$\underset{\_}{\text{C}}$SFSSAL$\underset{\_}{\text{C}}$VVH$\underset{\_}{\text{C}}$RVRGYPDGY$\underset{\_}{\text{C}}$SRKGI$\underset{\_}{\text{C}}$T$\underset{\_}{\text{C}}$RR	34	+5				
FT$\underset{\_}{\text{C}}$NSYA$\underset{\_}{\text{C}}$KAH$\underset{\_}{\text{C}}$ILQGHKSGS$\underset{\_}{\text{C}}$ARINL$\underset{\_}{\text{C}}$K$\underset{\_}{\text{C}}$QR	32	+5				
Defensin(*Drosophila melanogaster*)						Dimarcq et al., 1994
AT$\underset{\_}{\text{C}}$DLLSKWNWNHTA$\underset{\_}{\text{C}}$AGH$\underset{\_}{\text{C}}$IAKGFKGGY$\underset{\_}{\text{C}}$NDKAV$\underset{\_}{\text{C}}$V$\underset{\_}{\text{C}}$RN	40	+3	X	–	n.d.	
Navidefensin2-2(*Nasonia vitripennis*)						Gao and Zhu, 2010
FS$\underset{\_}{\text{C}}$DVLSFQSKWVSPNHSA$\underset{\_}{\text{C}}$AVR$\underset{\_}{\text{C}}$LAQRRKGGK$\underset{\_}{\text{C}}$KNGD$\underset{\_}{\text{C}}$V$\underset{\_}{\text{C}}$R	42	+5	X	–	–	
Defensin NV(*Nasonia vitripennis*)						Ye et al., 2010
VT$\underset{\_}{\text{C}}$ELLMFGGVVGDSA$\underset{\_}{\text{C}}$AAN$\underset{\_}{\text{C}}$LSMGKAGGS$\underset{\_}{\text{C}}$NGGL$\underset{\_}{\text{C}}$D$\underset{\_}{\text{C}}$RKTTFKELWDKRFG	52	+1	X	X	X	
Defensin(*Bombus pascuorum*)						Rees et al., 1997
VT$\underset{\_}{\text{C}}$DLLSIKGVAEHSA$\underset{\_}{\text{C}}$AAN$\underset{\_}{\text{C}}$LSMGKAGGR$\underset{\_}{\text{C}}$ENGI$\underset{\_}{\text{C}}$L$\underset{\_}{\text{C}}$RKTTFKELWDKRF^*^	51	+3	X	X	n.d.	
Royalisin(*Apis mellifera*)						Fujiwara et al., 1990
VT$\underset{\_}{\text{C}}$DLLSFKGQVNDSA$\underset{\_}{\text{C}}$AAN$\underset{\_}{\text{C}}$LSLGKAGGH$\underset{\_}{\text{C}}$EKGV$\underset{\_}{\text{C}}$I$\underset{\_}{\text{C}}$RKTSFKDLWDKRF^*^	51	+3	X	–	n.d.	
SpliDefensin(*Spodoptera littoralis*)						Seufi et al., 2011
VS$\underset{\_}{\text{C}}$DFEEANEDAV$\underset{\_}{\text{C}}$QEH$\underset{\_}{\text{C}}$LPKGYTYGI$\underset{\_}{\text{C}}$VSHT$\underset{\_}{\text{C}}$S$\underset{\_}{\text{C}}$IYIVELIKWYTNTYT	50	−5	X	X	–	
PxDef(*Plutella xylostella*)						Xu et al., 2016
RIP$\underset{\_}{\text{C}}$QYEDATEDTI$\underset{\_}{\text{C}}$QQH$\underset{\_}{\text{C}}$LPKGYSYGI$\underset{\_}{\text{C}}$VSYR$\underset{\_}{\text{C}}$S$\underset{\_}{\text{C}}$V	37	−1	X	X	X	
DLP4(*Hermetia illucens*)						Park et al., 2015
AT$\underset{\_}{\text{C}}$DLLSPFKVGHAA$\underset{\_}{\text{C}}$AAH$\underset{\_}{\text{C}}$IARGKRGGW$\underset{\_}{\text{C}}$DKRAV$\underset{\_}{\text{C}}$N$\underset{\_}{\text{C}}$RK	40	+6	X	–	n.d.	
Defensin 1(*Tribolium castaneum*)						Rajamuthiah et al., 2015
VT$\underset{\_}{\text{C}}$DLLSAEAKGVKVNHAA$\underset{\_}{\text{C}}$AAH$\underset{\_}{\text{C}}$LLKRKRGGY$\underset{\_}{\text{C}}$NKRRI$\underset{\_}{\text{C}}$V$\underset{\_}{\text{C}}$RN	44	+8	X	n.d.	n.d.	
Defensin(*Simulium bannaense*)						Wei et al., 2015
AT$\underset{\_}{\text{C}}$DLLSISTPWGSVNHAA$\underset{\_}{\text{C}}$AAH$\underset{\_}{\text{C}}$LALNRGFRGGY$\underset{\_}{\text{C}}$SSKAV$\underset{\_}{\text{C}}$T$\underset{\_}{\text{C}}$RK	46	+4	X	–	n.d.	
Defensin(*Cimex lectularius*)						Kaushal et al., 2016
AT$\underset{\_}{\text{C}}$DLFSFQSKWVTPNHAA$\underset{\_}{\text{C}}$AAH$\underset{\_}{\text{C}}$TARGNRGGR$\underset{\_}{\text{C}}$KKAV$\underset{\_}{\text{C}}$H$\underset{\_}{\text{C}}$RK	43	+7	X	–	n.d.	
Psdefensin(*Protaetia brevitaris seulensis*)						Lee et al., 2016
VT$\underset{\_}{\text{C}}$DLLSLQIKGIAINDSA$\underset{\_}{\text{C}}$AAH$\underset{\_}{\text{C}}$LAMRRKGGS$\underset{\_}{\text{C}}$KQGV$\underset{\_}{\text{C}}$V$\underset{\_}{\text{C}}$RN	43	+4	X	X	n.d.	
Defensin(*Oryctes rhinoceros*)						Ishibashi et al., 1999
LT$\underset{\_}{\text{C}}$DLLSFEAKGFAANHSL$\underset{\_}{\text{C}}$AAH$\underset{\_}{\text{C}}$LAIGRKGGA$\underset{\_}{\text{C}}$QNGV$\underset{\_}{\text{C}}$V$\underset{\_}{\text{C}}$RR	43	+3	X	n.d.	n.d.	
Defensin A, B(*Anomala cuprea*)						Yamauchi, 2001
VT$\underset{\_}{\text{C}}$DLLSFEAKGFAANHSI$\underset{\_}{\text{C}}$AAH$\underset{\_}{\text{C}}$LAIGRKGGS$\underset{\_}{\text{C}}$QNGV$\underset{\_}{\text{C}}$V$\underset{\_}{\text{C}}$RN	43	+2	X	–	n.d.	
VT$\underset{\_}{\text{C}}$DLLSFEAKGFAANHSI$\underset{\_}{\text{C}}$AAH$\underset{\_}{\text{C}}$LVIGRKGGA$\underset{\_}{\text{C}}$QNGV$\underset{\_}{\text{C}}$V$\underset{\_}{\text{C}}$RN	43	+2	X	X	n.d.	
Defensin(*Calliphora vicina*)						Chernysh et al., 2000
AT$\underset{\_}{\text{C}}$DLLSGTGANHSA$\underset{\_}{\text{C}}$AAH$\underset{\_}{\text{C}}$LLRGNRGGY$\underset{\_}{\text{C}}$NGKAV$\underset{\_}{\text{C}}$V$\underset{\_}{\text{C}}$RN	40	+3	X	–	–	
Holotricin(*Holotrichia diomphalia*)						Lee et al., 1995
VT$\underset{\_}{\text{C}}$DLLSLQIKGIAINDSA$\underset{\_}{\text{C}}$AAH$\underset{\_}{\text{C}}$LAMRRKGGS$\underset{\_}{\text{C}}$KQGV$\underset{\_}{\text{C}}$V$\underset{\_}{\text{C}}$RN	43	+4	X	–	n.d.	
PduDefensin^f^ (*Phlebotomus duboscqi*)						Boulanger et al., 2004
AT$\underset{\_}{\text{C}}$DLLSAFGVGHAA$\underset{\_}{\text{C}}$AAH$\underset{\_}{\text{C}}$IGHGYRGGY$\underset{\_}{\text{C}}$NSKAV$\underset{\_}{\text{C}}$T$\underset{\_}{\text{C}}$RR	40	+3	X	n.d.	X	
Defensin(*Pyrrhocoris apterus*)						Cociancich et al., 1994
AT$\underset{\_}{\text{C}}$DILSFQSQWVTPNHAG$\underset{\_}{\text{C}}$ALH$\underset{\_}{\text{C}}$VIKGYKGGQ$\underset{\_}{\text{C}}$KITV$\underset{\_}{\text{C}}$H$\underset{\_}{\text{C}}$RR	43	+4	X	X	n.d.	
Defensin(*Palomena prasina*)						Chernysh et al., 1996
AT$\underset{\_}{\text{C}}$DALSFSSKWLTVNHSA$\underset{\_}{\text{C}}$AIH$\underset{\_}{\text{C}}$LTKGYKGGR$\underset{\_}{\text{C}}$VNTI$\underset{\_}{\text{C}}$N$\underset{\_}{\text{C}}$RN	43	+4	X	X	–	
Coprisin^c^ (*Copris tripartitus*)						Hwang et al., 2009; Lee et al., 2012
VT$\underset{\_}{\text{C}}$DVLSFEAKGIAVNHSA$\underset{\_}{\text{C}}$ALH$\underset{\_}{\text{C}}$IALRKKGGS$\underset{\_}{\text{C}}$QNGV$\underset{\_}{\text{C}}$V$\underset{\_}{\text{C}}$RN	43	+3	X	n.d.	X	
Defensin B, C(*Zophobas atratus*)						Bulet et al., 1991
FT$\underset{\_}{\text{C}}$DVLGFEIAGTKLNSAA$\underset{\_}{\text{C}}$GAH$\underset{\_}{\text{C}}$LALGRRGGY$\underset{\_}{\text{C}}$NSKSV$\underset{\_}{\text{C}}$V$\underset{\_}{\text{C}}$R	43	+3	X	X	n.d.	
FT$\underset{\_}{\text{C}}$DVLGFEIAGTKLNSAA$\underset{\_}{\text{C}}$GAH$\underset{\_}{\text{C}}$LALGRTGGY$\underset{\_}{\text{C}}$NSKSV$\underset{\_}{\text{C}}$V$\underset{\_}{\text{C}}$R	43	+2	X	X	n.d.	
Defensin 1(*Acalolepta luxuriosa*)						Ueda et al., 2011
FT$\underset{\_}{\text{C}}$DVLSVEAKGVKLNHAA$\underset{\_}{\text{C}}$GIH$\underset{\_}{\text{C}}$LFRRRTGGY$\underset{\_}{\text{C}}$NKKRV$\underset{\_}{\text{C}}$I$\underset{\_}{\text{C}}$R	43	+7	X	X	n.d.	
Defensin A(*Chironomus plumosus*)						Lauth et al., 1998
LT$\underset{\_}{\text{C}}$DILGSTPA$\underset{\_}{\text{C}}$AAH$\underset{\_}{\text{C}}$IARGYRGGW$\underset{\_}{\text{C}}$DGQSV$\underset{\_}{\text{C}}$N$\underset{\_}{\text{C}}$RR	36	+2	X	–	n.d.	
Mdde(*Musca domestica*)						Wang et al., 2006
AT$\underset{\_}{\text{C}}$DLLSGTGVGHSA$\underset{\_}{\text{C}}$AAH$\underset{\_}{\text{C}}$LLRGNRGGY$\underset{\_}{\text{C}}$NGKGV$\underset{\_}{\text{C}}$V$\underset{\_}{\text{C}}$RN	40	+3	X	X	–	
Defensin(*Formica rufa*)						Taguchi et al., 1998
FT$\underset{\_}{\text{C}}$DLLSGAGVDHSA$\underset{\_}{\text{C}}$AAH$\underset{\_}{\text{C}}$ILRGKTGGR$\underset{\_}{\text{C}}$NSDRV$\underset{\_}{\text{C}}$V$\underset{\_}{\text{C}}$RA	40	+2	X	n.d.	n.d.	

a Conserved cysteines are underlined in green for ease of comparison,

b G+/G− gram-positive/gram-negative bacteria, F filamentous fungi, tested activity of peptides is indicated by X (active) or — (inactive) or n.d., if not determined, activities are extracted from given references as well as the Defensin Knowledgebase (Seebah et al., 2007),

c NMR structure has been resolved,

d Asterisk indicates C-terminal amidation,

e Active against Trypanosoma brucei,

f *Active against Leishmania major*.

## Structure-activity relationships of insect defensins

The variety of insect AMPs is also reflected within their secondary and tertiary structures. They can be broadly divided into three major groups, i.e., (i) primarily α-helical peptides, (ii) peptides rich in one particular amino acid (e.g., proline or glycine) and (iii) cysteine-rich peptides. Recent review articles have addressed current knowledge about groups (i) and (ii) (Huang et al., 2010; Li et al., 2014; Xhindoli et al., 2016). Based on additional secondary structure elements cysteine-rich insect AMPs can be subdivided into, (i) peptides exhibiting antiparallel triple-stranded β-sheets (e.g., Alo-3; Barbault et al., 2003), (ii) peptides that form a hairpin-like β-sheet structure (e.g., thanatin; Mandard et al., 1998) and (iii) defensins, i.e., peptides with a complex arrangement of α-helixes and β-sheets stabilized by disulfide-bonds (e.g., phormicin; Cornet et al., 1995), which are focus of this review. Insect defensins are defined to contain six conserved cysteines that form a typical arrangement of three disulfide bonds. However, peptides such as drosomycin contain eight cysteines, which is a conserved feature of plant defensins. Further, these peptides consist of a α-helix and antiparallel β-sheets (Figures 1D–F). Two disulfide bonds connect the C-terminal β-sheet and the α-helix and the third connects the N-terminal loop with the second β-sheet. Similar to peptides from plants or fungi they are hence classified as *cis*-defensins as opposed to *trans*-defensins found within vertebrate species (Shafee et al., 2017). The tight arrangement of secondary structural elements is reflected in high stability against heat or proteases. Accordingly, this structural topology is known as cysteine-stabilized αβ motif (CSαβ) and is common among defensin peptides across different organisms, from plants to invertebrates to vertebrates (Dias Rde and Franco, 2015; Tarr, 2016; Shafee et al., 2017). Although all insect defensins share this common structural motif their primary sequence (Figure 1A) as well as their spectrum of antimicrobial activity varies considerably (Table 1). It is evident that the majority of tested peptides exhibits activity against gram-positive bacteria, however several peptides exhibit potent activity against gram-negative bacteria or are primarily active against fungi (Table 1). Repositories such as Defensin Knowledgebase or ADP (Seebah et al., 2007; Wang et al., 2016) are useful resources for retrieving sequences for activity comparisons.

Yet, the attempt to accurately compare antimicrobial activities and relate them to the peptide sequences and secondary structures is challenging. Importantly, there is a large variety of different pathogens that have been selected for testing of defensin activity and some peptides have only been tested for individual pathogens, e.g., only one single strain of a gram-positive or gram-negative bacterium and it is also worth to mention that testing for antifungal activity was not carried out in a large number of studies (Table 1). Thus, activity spectra for these peptides need yet to be established. Further, assay conditions and concentration thresholds that are used to describe peptides as active or inactive can vary remarkably. For example in the initial study that identified the specific antifungal activity of drosomycin the highest concentration tested was 20 μM and the peptide thus referred as inactive against bacteria (Fehlbaum et al., 1994). In contrast the termite-specific termicin was reported to have weak activity against bacteria only at concentrations between 25 and 100 μM (Lamberty et al., 2001b). Additionally a range of different ways to describe activity is used such as μg/mL, molarity or the diameter of growth inhibition at a given concentration, thus making a quick and direct comparison not trivial. When characterizing defensin activity it also has to be noted that some studies use AMPs devoid of cysteines as control peptides and such studies are more difficult to use for comparison. Not at least experimental conditions such as the use of varying salt concentrations can change the activity of individual peptides dramatically and should also be considered in activity comparisons (Lee et al., 1997).

With regard to structure-activity relationships, a key limitation for insect defensins is the low number of resolved three-dimensional structures. Comparisons purely based on primary sequences are error-prone (Grishin, 2001) and conservation within secondary and tertiary structure is higher as compared to the primary sequences (Shafee et al., 2016). Currently only nine peptides have been characterized using solution NMR spectroscopy, including four antifungal, i.e., heliomicin (Lamberty et al., 2001a), drosomycin (Landon et al., 1997), termicin (Da Silva et al., 2003), and ARD1 (Landon et al., 2004) as well as five anti-gram-positive peptides, i.e., phormicin (Cornet et al., 1995), sapecin (Hanzawa et al., 1990), Def-AAA (Landon et al., 2008), lucifensin (Nygaard et al., 2012), and coprisin (Lee et al., 2013). Nevertheless, these studies provide valuable information about structure-activity relations for both antibacterial as well as antifungal insect defensins and shed light on structural determinants underlying biological activity.

For example mutation studies on the antifungal peptide ARD1 revealed subtle changes in hydrophobicity and cationicity to enhance the activity spectrum and increase potency (Landon et al., 2004). In an attempt to confer anti-bacterial activity onto the antifungal heliomicin which only differs from ARD1 in two positions changes within the N-terminal sequence led to a loss of antifungal activity highlighting its functional importance (Lamberty et al., 2001a). It should be noted here that peptides with antifungal activity have a longer N-terminus that forms an additional β-sheet and brings N- and C-terminal residues in close proximity as compared to antibacterial counterparts (Figure 1F, Table 1). The third antifungal peptide with the length of 44 amino acids is drosomycin. Interestingly, it has an additional disulfide-bond similar to the plant defensins RsAfp2 or NaD1 (Van Der Weerden et al., 2013). Compared to the other antifungal insect defensins it has an additional disulfide bond that connects the N-terminal loop to the C-terminus of the peptide. A modeling study comparing drosomycin to other plant antifungal defensins such as RsAFP2 suggested a hydrophobic patch in which a lysine residue is embedded as key determinant for antifungal activity (Landon et al., 2000). Indeed, experimental evidence verified this lysine residue while testing the functional role of charged residues for the antifungal activity of drosomycin (Zhang and Zhu, 2010). The fourth antifungal insect defensin for which a structure has been resolved is the termite-specific termicin. It carries an overall net charge of +4 and seems to be overall less different to antibacterial defensins. While exhibiting an amphiphilic character similar to drosomycin or heliomicin, the positions of hydrophilic and hydrophobic residues exposed on the surface are opposite. Several residues including for example the two arginine residues in loop 3 were proposed as possible interacting partners involved in antifungal activity (Da Silva et al., 2003), however experimental evidence for this hypothesis is still missing. The other three antifungal defensins known to date are Gallerimycin, Gm defensing, and Gm defensin-like peptide (Schuhmann et al., 2003; Cytrynska et al., 2007). Gm defensin and Gm defensin-like peptide show the highest sequence similarity (~90% identity) to heliomicin and ARD1 including a conserved N-terminal sequence stretch, whereas Gallerimycin has a particular long N-terminus and shorter loop 1 and loop 5 sequences (Table 1). It appears that multiple factors contribute to specificity toward antifungal activity involving the N-terminal portion of the peptide as well as a subtle interplay between hydrophobic and charged residues.

For the primarily antibacterial defensins only five available structures represent a very limited number given the large number of different peptide sequences (Table 1). Additionally it is worth to mention that phormicin, sapecin, and lucifensin only differ by individual amino acids and thus it is not surprising that their three-dimensional topologies are highly similar (Hanzawa et al., 1990; Cornet et al., 1995; Nygaard et al., 2012). In an attempt to increase activity against *Staphylococcus aureus* a detailed study was reported using the *Anopheles* defensin as well as an alignment of 40 insect defensin sequences as basis for the design of 45 peptide mutants (Landon et al., 2008). A change in loop 1 (-GFGVGSSL- to -KWNWHTA-) resulted in a peptide with increased activity but also increased toxicity as compared to the native defensin. A second series of mutations further underpinned the importance of loop 1 for both, activity against *S. aureus* as well as toxicity yet fails to identify single residues that are responsible and highlighting the complexity of sequence-based approaches. Although sequence differences in loop 1 were clearly reflected by differences in the three-dimensional structures, all but one peptide were found to have the CSαβ fold. This is in line with the sequence of the dung-beetle defensin coprisin. It has an elongated loop 1 sequence that shows a high degree of flexibility along the typical well-defined CSαβ portion (Lee et al., 2013) that is similar to all other insect defensins. A follow-up study that investigated the importance of the disulfide-bonds within coprisin clearly demonstrated that a removal of cysteine residues leads to dramatic loss of activity against bacteria, whereas antifungal activity was less affected (Lee et al., 2014). Notably also a series of non-apeptides showed significantly reduced activity and thus underpins the importance of the CSαβ motif for antimicrobial activity.

In addition to these factors relating to three-dimensional aspects of insect defensins, it is unclear whether posttranslational modifications such as amidation do play a role with regard to peptide activity. Amidation is common upon many bioactive peptides and often crucial for activity. It is also found within insect AMPs such as for example cecropins (Steiner et al., 1981). However, most of the insect defensins known to date are described as C-terminal acids and only few examples such as the bumblebee defensin or the termite defensin termicin have been identified by mass spectrometry to be C-terminally amidated (Rees et al., 1997; Lamberty et al., 2001b; Favreau et al., 2006). Possible implications regarding the biological activity and mechanism-of-action remain speculative.

## Mechanisms-of-action of insect CSαβ peptides—from membrane effects to ion channel pharmacology

Similar to the broad range of microorganisms targeted by insect defensins their mechanisms-of-action seem equally complex and knowledge remains sparse. Their activity is primarily explained by the presence of positive charges exposed on the surface (Figures 1E,F) that can interact with negative charges on the outside of microbes leading to the insertion and perforation of membranes (Brogden, 2005). However, it is nowadays evident that antimicrobial peptides are also acting on specific target structures such as lipid II or sphingolipids (Wilmes et al., 2011). To date specific protein targets for insect defensins remain to be identified and structure-activity studies may prove useful in deciphering molecular mechanism underlying bioactivity. Further, reported activity of anionic antimicrobial peptides (Xu et al., 2016) clearly indicates that electrostatic interactions alone cannot explain the antimicrobial activity, and it remains to be shown if these peptides exhibit different mechanisms-of-actions. Interestingly there are several insect defensin-like CSαβ peptides from scorpions that potently inhibit voltage gated potassium channels. Indeed there seems to be an evolutionary link between the antimicrobial and (neuro)-toxic activity of peptides containing a CSαβ fold (Zhu et al., 2014). From an evolutionary perspective it seems evident that the presence of a conserved three-dimensional fold in both antimicrobial defense peptides and scorpion toxins reflects a common strategy to defend against invading organism or predators by means of membrane interaction. Within scorpion toxins a conserved lysine residue interacting with the channel's selectivity filter characterizes the pore-blocking activity (Garcia et al., 2001). Although this residue is also found in some insect defensins, it appears that a flexible N-terminal loop (=loop 1) within antimicrobial defensins impairs access to the channel pore as compared to scorpion type defensins that lack such a loop (Zhu et al., 2014). Notably this intercysteine loop sequence shows high variability (*n* = 4–16, Figure 1A) and thus it allows speculations regarding potential promiscuous activity of individual insect peptides. Though, to date no study attempted to accurately analyse this evolutionary link of antimicrobial insect peptides and scorpion K_v_ toxins and it is still unclear how peptides that share a such a high degree of structural similarity can exhibit such a diverse range of biological activities.

## Conclusion

Given the number of insect species, the variety of defensin peptides and a range of activities it is evident that insect defensins are valuable structural templates for rational design of a novel class of “designer AMPs.” Hence it is crucial to have a detailed understanding on how structural aspects are reflected in peptide activity both with regard to pathogen specificity as well as undesired side effects such as cell toxicity. Current literature provides a solid, yet incomplete basis for rational structure based drug design. The urgent need for the development of novel antibiotic lead molecules provides significant justification and new impetus for further detailed exploration of structure-activity relationships of antimicrobial insect CSαβ peptides.

## Author contributions

The author confirms being the sole contributor of this work and approved it for publication.

### Conflict of interest statement

The author declares that the research was conducted in the absence of any commercial or financial relationships that could be construed as a potential conflict of interest.
